# Research opportunities in Classical Hematology

**DOI:** 10.1016/j.htct.2024.10.001

**Published:** 2024-11-30

**Authors:** Carla Roberta Peachazepi Moraes, Edwin Mayr, Erich Vinicius De Paula

**Affiliations:** a Faculdade de Ciências Médicas, Universidade de Campinas (FCM Unicamp), Campinas, SP, Brazil; b Hemocentro, Universidade de Campinas (FCM Unicamp), Campinas, SP, Brazil

Classical Hematology (CH) is a term used to define a field that encompasses non-malignant hematological diseases such as congenital and acquired non-clonal anemias and hemostatic disorders. In the past this field has been extensively referred to as “benign” or “general” hematology. However, these terms were replaced by CH based on the fear that language such as benign and general could dismiss the complexity and severity of such conditions, thus reducing interest in the field, for both researchers and hematologists.^1^

Despite the inherent relevance of the field, the number of exclusive classical hematology clinicians in comparison to those dedicated to onco-hematology is alarmingly low. A survey conducted by the American Society of Hematology (ASH) in 2018 demonstrated that only 4-5% of adult hematology-oncology fellows present an interest in specializing in CH.^2^ Furthermore, an analysis of hematology-oncology fellowship program websites that were accredited by the Accreditation Council for Graduate Medical Education (ACGME) indicated that out of the 172 available websites from September 2021 to January 2022, only 57 (33%) mentioned the term “Classical Hematology”.^3^

The potential reasons for this phenomenon may involve several underlying causes such as: lack of adequate mentorship, content and quality of medical fellowship, lack of adequate funding, job availability and financial prospects.^2^ Mentorship has widely been regarded as one of the most relevant factors influencing CH retention. Several studies found interesting connections between a positive mentoring relationship and an increase in pursuing CH as a career, as well an impact in personal and professional identity ^4^. In contrast, the negative effect of a lack of classical hematologists manifests as a shortage of available mentors for trainees interested in the field.^5^ To address these challenges, the development of targeted and specialized fellowship and mentorship programs in CH, with opportunities to interact with patients and engage in research activities can be crucial for shaping potential career paths.^6^

In Brazil, there is no available data on the proportion of hematologists dedicated to CH, nor on the challenges faced in training for this area. However, it is our perception that at least when it comes to professional prospects, onco-hematology attracts more clinicians than CH, even though a recent survey of nearly 100 former hematology residents from a Brazilian academic institution confirmed the importance of CH in clinical practice.^7^

In this context, this issue of "*Hematology, Transfusion and Cell Therapy*" emerges as an encouraging counterpoint to these arguments, as it showcases more than 40 articles addressing contemporary issues related to CH, and confirming the dynamism and research opportunities in the field. In fact, areas such as transfusion medicine, congenital anemias, congenital and acquired bleeding disorders, as well as emerging fields such as implementation science, health service evaluation, medical education among others have long represented opportunities for basic, translational and clinical research, sometimes opening important paths for research in oncology and other areas of healthcare.
